# Diversity of Mycotoxins in Stored Paddy Rice: Contamination Patterns in the Mekong Delta, Vietnam

**DOI:** 10.3390/toxins17010006

**Published:** 2024-12-26

**Authors:** Lien Thi Kim Phan, Thuy Thi Ngoc Nguyen, Thien Thi Thanh Tran, Sarah De Saeger

**Affiliations:** 1 Faculty of Food Science and Technology, Ho Chi Minh City University of Industry and Trade, 140 Le Trong Tan Street, Tay Thanh Ward, Tan Phu District, Ho Chi Minh City 70000, Vietnam; thuyntn@huit.edu.vn (T.T.N.N.); thanhthien1512pt@gmail.com (T.T.T.T.); 2 Mytox-South^®^, International Thematic Network, Ghent University, 9000 Ghent, Belgium; sarah.desaeger@ugent.be; 3 Department of Bioanalysis, Faculty of Pharmaceutical Sciences, Ghent University, 9000 Ghent, Belgium

**Keywords:** diversity, emerging mycotoxins, evaluation, Mekong Delta, contamination, stored paddy rice

## Abstract

Rice (*Oryza sativa* L.) is the most important food in Vietnam. However, rice is often lost in post-harvest due to fungal growth and mycotoxins contamination. This study aimed to evaluate mycotoxin contamination in stored paddy rice collected in 2018, 2019, and 2022 in six provinces in Mekong Delta, Vietnam, using LC-MS/MS. The results revealed that 47% of the samples were contaminated with 12 types of mycotoxins. The prevalence of these mycotoxins was 30% (ZEN), 10% (FUS/MON), 6% (BEA/AFB2), 2–4% (AFG1, AFB1, AFG2), 2% (FB1), and 1% (OTA/AME/ENB). Among the provinces, stored paddy rice from Kien Giang had the highest contamination, followed by Ben Tre, Long An, An Giang, Dong Thap, and Can Tho. Remarkably, paddy rice collected in 2022 was usually contaminated with emerging mycotoxins with a higher incidence of co-occurrence ranging from 2–6% of the samples. Additionally, five stored paddy rice samples were contaminated with levels of AFB1, OTA, and ZEN exceeding Vietnamese regulatory limits for unprocessed rice. Our findings provide valuable insights into mycotoxin contamination across different years and growing regions in the Mekong Delta, Vietnam. This study could give essential information to stakeholders, including policy-makers or food safety authorities, etc., to inform strategies to mitigate these toxins in the near future and underscores the importance of monitoring rice production.

## 1. Introduction

Rice (*Oryza sativa* L.) is one of the most important staple foods worldwide, feeding approximately 75% of the global population [1]. It is particularly vital for the majority of people in Asia [2]. Rice is typically stored as paddy rice for several months or even years (e.g., in India) [2]. To produce brown rice, paddy rice is de-hulled, and to produce polished or white rice, the bran layer of brown rice is removed [3,4,5]. Rice is primarily cultivated in sub-tropical and tropical regions with warm and humid conditions [1]. In practice, rice is generally dried and stored under poor technical conditions and/or in inappropriate facilities post-harvest, fostering an ideal environment for fungal proliferation in most developing countries, such as Vietnam [5], especially in high-moisture and high-temperature conditions such as India [6]. Approximately 15% of harvested rice is lost annually due to improper storage conditions, which leads to fungal growth [7]. Furthermore, rice is often contaminated with mycotoxins, such as aflatoxins (AFs) in rice from Vietnam [8]. The temperature and moisture conditions prevalent during storage promote AF production, impacting the economies of rice-producing countries [9,10,11]. AFs are classified as Group 1 carcinogens (carcinogenic to humans), contributing to 5–28% of global hepatocellular carcinoma (HCC) cases [11]. *Aspergillus flavus*, an AF-producing fungus [12,13], often proliferates in post-harvest stages, particularly during storage [14,15]. Fumonisins (FBs) are classified as Group 2B carcinogens (possibly carcinogenic to humans) [16]. FB-producing fungi, known as field pathogens of crops, include *Fusarium verticillioides*, a major producer [12,13]. Zearalenone (ZEN), mainly produced by *Fusarium graminearum* and *Fusarium culmorum*, is commonly found in rice. Although ZEN exhibits relatively low acute toxicity, its chronic effects are a serious concern [4]. Ochratoxin A (OTA) is the most toxic mycotoxin within the ochratoxin group. It is typically produced by *Penicillium verrucosum* and *Aspergillus ochraceus* and is classified as a Group 2B carcinogen (possibly carcinogenic to humans) [16]. OTA has been shown to be nephrotoxic, teratogenic, immunotoxic, and carcinogenic to humans. Emerging mycotoxins such as beauvericin (BEA), enniatin B (ENB), fusaric acid (FUS), and moniliformin (MON) are lesser-known toxins. These mycotoxins are principally produced by various *Fusarium* species and commonly occur in cereals. BEA and ENB are cytotoxic to human white blood cell progenitors, platelet progenitors, and red blood cell progenitors. MON has been shown to cause chromosomal aberrations [17].

Certain environmental factors influence the occurrence of fungi and mycotoxins in rice. The severity of contamination depends on geographical location, agricultural practices, and the susceptibility of commodities to fungal invasion during pre-harvest, storage, and processing [18,19,20]. Hence, many countries have implemented regulations to limit exposure to mycotoxins by setting maximum allowable limits in food and feed [21,22]. In Vietnam, the maximum limits for AFB1 and total AFs in unprocessed rice are set at 5 µg/kg and 10 µg/kg, respectively, while the limits for OTA and ZEN in unprocessed cereals are 5 µg/kg and 100 µg/kg, respectively [23]. However, no maximum limits have been established for other toxins, such as BEA, MON, ENB, and FUS, in Vietnamese food.

Rice is cultivated across Vietnam, including the Northeast, Red Delta, North-Central Coast, and Mekong Delta regions. The Mekong Delta accounts for more than 50% of the country’s total rice output and over 90% of its rice exports. In this region, rice is primarily cultivated in Can Tho, Dong Thap, An Giang, Kien Giang, Tien Giang, Ben Tre, and Long An [24]. Vietnam is a tropical country, with an average temperature range of 18–31 °C. Specifically, the average temperatures in the Mekong Delta in 2018, 2019, and 2022 ranged from 26.6 to 29.3 °C, 26.5 to 29.9 °C, and 26.6 to 28.7 °C, respectively. Annual rainfall in these years ranged from 0.2 to 523 mm, 0.8 to 465 mm, and 0.1 to 565 mm, respectively, with an average relative humidity between 75% and 86% [24]. The warm and humid weather conditions in Vietnam are conducive to fungal growth and, consequently, mycotoxin production. Although a previous study examined the impacts of environmental conditions and agricultural practices on mycotoxin contamination in the Mekong Delta’s paddy rice supply chain, it focused solely on AF and FB contamination in three provinces [5]. It did not assess the presence of various mycotoxins in stored paddy rice across different rice-growing regions [5]. Therefore, this study aimed to evaluate mycotoxin contamination in stored paddy rice over three years across six provinces in the Mekong Delta region, Vietnam, to assess the potential risk of contamination and determine whether storage conditions increase its likelihood.

## 2. Results

### 2.1. Mycotoxin Contamination in Stored Paddy Rice Collected in 2018, 2019 and 2022

In this study, 96 stored paddy rice samples were collected from warehouses of rice companies and farmers in the Mekong Delta region during 2018 (n = 24), 2019 (n = 22), and 2022 (n = 50), and the presence of mycotoxins was analyzed using LC-MS/MS. The results revealed that 47% of the samples collected over three years were contaminated with 12 types of mycotoxins. The prevalence of ZEN was highest (30%) while the values of all others were lower than 10%.

**Figure 1:**
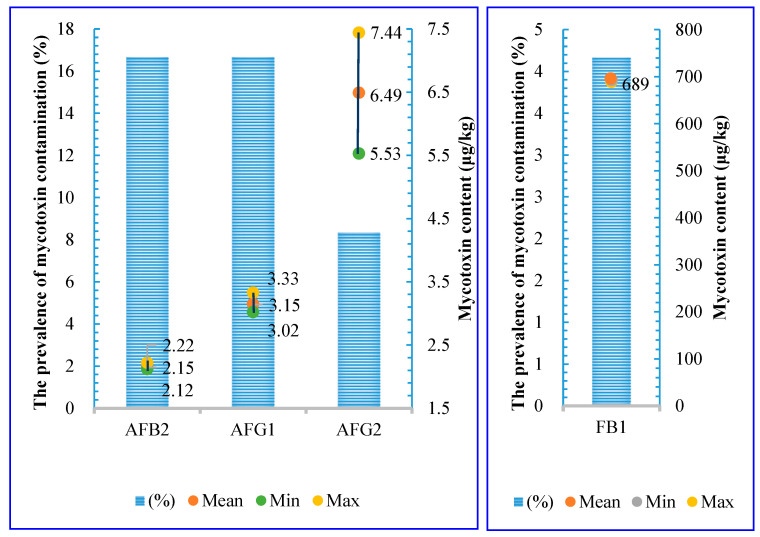
Prevalence (%) and mycotoxins concentration (µg/kg) in stored paddy rice in 2018 (AFB1: Aflatoxin B1; AFB2: Aflatoxin B2; AFG2: Aflatoxin G2; FB1: Fumonisin B1).

In detail, 37.50% (n = 9/24) of the samples collected in 2018 were contaminated with AFs and FBs, with contamination frequencies of 33% and 4%, respectively (Figure 1). Specifically, the prevalence of AFB2/AFG1, AFG2, and FB1 was 17%, 8%, and 4%, respectively. Additionally, two samples (8.33%) were co-contaminated with AFG1 and AFG2. The average total aflatoxin content was 4.27 ± 3.37 µg/kg. The mean levels of AFB2, AFG1, AFG2, and FB1 were mentioned in Figure 1. Importantly, no samples were contaminated with levels of mycotoxins exceeding Vietnamese regulation levels.

**Figure 2:**
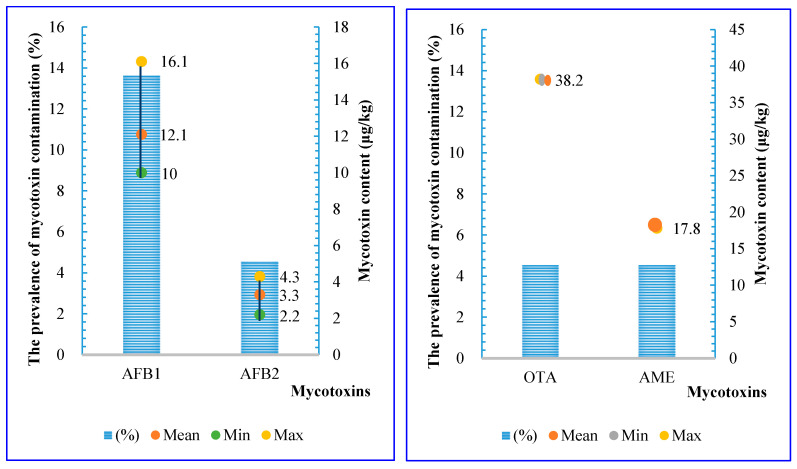
Prevalence (%) and mycotoxins concentration (µg/kg) in stored paddy rice in 2019 (AFB1: Aflatoxin B1; AFB2: Aflatoxin B2; OTA: Ochratoxin A; AME: Alternariol Monomethyl Ether).

As shown in Figure 2, 36% (n = 8/22) of the samples collected in 2019 were contaminated with at least one mycotoxin, with 9% of the mycotoxin-positive samples showing simultaneous contamination with more than one toxin. OTA, AFB1, AFB2, and AME were detected in these samples, whereas AFG1, AFG2, and FB1, which were found in the 2018 samples, were not detected in 2019 (Figure 2). Notably, AFB1 had the highest prevalence (14%) compared to AFB2, OTA, and AME (5%). The mean concentrations of these mycotoxins ranged from 3.30 ± 1.5 to 38.20 µg/kg. All positive samples contaminated with AFB1 and OTA exceeded Vietnamese regulation levels for unprocessed rice (5 µg/kg) [23].

**Figure 3:**
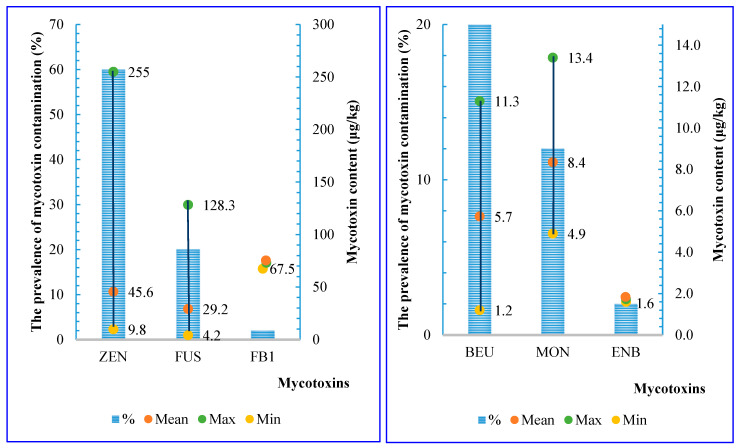
The prevalence (%) and mycotoxins concentration (µg/kg) in stored paddy rice in 2022 (ZEN: Zearalenone; FUS: Fusaric acid; FB1: Fumonisin B1; BEA: Beauvericin; MON: Moniliformin; ENB: Enniatin B).

Based on the data, 70% of the samples collected in 2022 (n = 35/50) were contaminated with at least one of the emerging mycotoxins. These samples were contaminated with six types of mycotoxins, as shown in Figure 3. Notably, three types of mycotoxins, ZEN, BEA, and ENB, were detected in 2022 that were not observed in the 2018 and 2019 samples (Figure 1 and Figure 2). Among these, ZEN had the highest prevalence, followed by FUS, BEA, etc. The range of average concentrations of these mycotoxins were from 5.7 ± 4.0 to 67.5 µg/kg.

Regarding co-contamination of mycotoxins in these samples, the results indicated that 48% of the samples were contaminated with only one mycotoxin, while 2% to 6% of the samples were contaminated with two to five types of mycotoxins. As shown in Table 1, some emerging mycotoxins frequently co-occurred in the same sample, with combinations including two mycotoxins (FUA-BEA/ZEN), three mycotoxins (ZEN/MON-FUA-BEA), four mycotoxins (ZEN-FUA-BEA-ENB/MON), and five mycotoxins (ZEN-FUA-BEA-MON-FB1).

### 2.2. Mycotoxins Contamination in Stored Paddy Rice Collected in Different Regions

**Figure 4:**
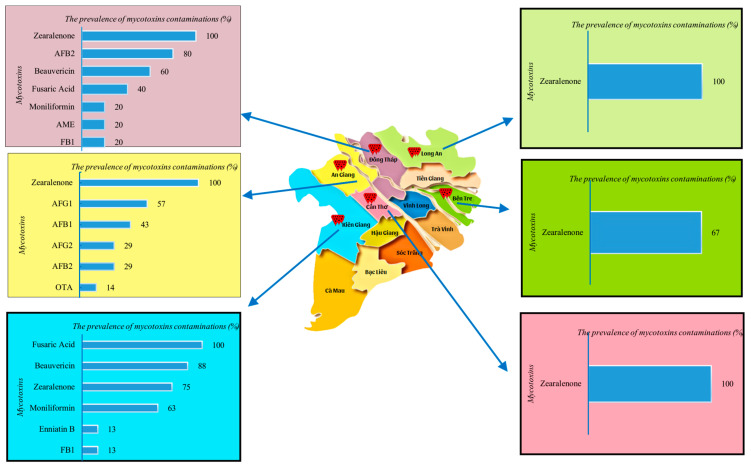
The prevalence (%) of mycotoxin contaminations in stored paddy rice collected in different regions in Mekong Delta, Vietnam. (AFG1: Aflatoxin G1; AFG2: Aflatoxin G2; AFB1: Aflatoxin B1; AFB2: Aflatoxin B2; OTA: Ochratoxin A; FB1: Fumonisin B1; AME: Alternariol Monomethyl Ether).

Analysis of mycotoxins in 96 stored paddy rice samples collected from the Mekong Delta revealed a variety of incidences of contamination across provinces. The samples were collected from Kien Giang (n = 10), Ben Tre (n = 3), Long An (n = 8), An Giang (n = 24), Dong Thap (n = 25), and Can Tho (n = 26). The results showed that Kien Giang had the highest contamination (80%), followed by Ben Tre and Long An, whereas contamination in other provinces was lower (60%) (Figure 4).

In detail, seven types of mycotoxins were detected in stored paddy rice from Dong Thap. ZEN was found in all observed samples (100%), followed by AFB2 and BEA, etc. (Figure 4). The mean concentration of ZEN was 82.5 ± 97.2 µg/kg, with a maximum level of 255 µg/kg (Table 2), exceeding the maximum limit of 100 µg/kg set by Vietnamese regulations [23]. A high range of mean mycotoxin concentrations was estimated in this province, ranging from 2.2 ± 0.1 to 66.3 ± 87.8 µg/kg (Table 2). Only one sample was contaminated with FB1 and MON. Notably, 8% of the samples were co-contaminated with three mycotoxins (MON/ZEN-FUS-BEA).

Several emerging mycotoxins (FUS, BEA, ZEN, MON, ENB) and FB1 were also frequently found in Kien Giang’s paddy rice (Figure 4 and Table 2), showing similarities to the mycotoxins identified in Dong Thap. Among these, FUS was detected in all samples (100%), and BEA, ZEN, and MON were found in 63% to 88% of the samples. ENB and FB1 were detected at the lowest frequencies (13%). Notably, several samples were co-contaminated with multiple mycotoxins, as mentioned in Table 1, with an incidence ranging from 8% to 30%. The average levels of ZEN, FB1, and FUS were relatively high, ranging from 19.9 ± 15.2 to 67.5 µg/kg, whereas the concentrations of other toxins were lower. Importantly, no samples exceeded the maximum limits for mycotoxins in food as specified by Vietnamese regulations [23].

Remarkably, six types of mycotoxins were detected in paddy rice from An Giang (Figure 4 and Table 2). ZEN was present in 100% of the samples, with a mean concentration of 27.8 ± 14.4 µg/kg. Additionally, several mycotoxins found in An Giang’s paddy rice were not detected in other provinces. Specifically, four types of aflatoxins were identified, ranging from 29 to 57% of samples. The average levels of aflatoxins ranged from 3.1 ± 0.1 to 12.1 ± 3.5 µg/kg. Notably, three samples were contaminated with AFB1 with levels of 10 to 16.1 µg/kg, and one sample was contaminated with OTA at 38.2 µg/kg, which are much higher than the maximum limits for AFB1 and OTA in food (5 µg/kg) established by Vietnamese regulations [23].

Amazingly, stored paddy rice collected from Long An, Ben Tre, and Can Tho was only contaminated by ZEN, with an average content of 22.8 ± 3.7 to 67.5 ± 56 µg/kg (Table 2) and a high prevalence of 67 to 100% (Figure 4). One sample collected in Long An exceeded the Vietnamese maximum limit (100 µg/kg) [19].

## 3. Discussions

Rice, along with wheat and corn, is a major staple food in many countries [25]. It contributes approximately 27% of the global dietary energy supply and 20% of dietary protein consumption [26,27]. However, rice is often harvested at very high moisture levels (35–50%) [5,25,28]. Therefore, mycotoxin-producing fungi could infect the grain, which can produce mycotoxins during storage [5].

Twelve types of mycotoxins identified in this study align with those reported in several previous publications. Specifically, the contamination patterns of AFs, including AFB2, AFG1, and AFG2, in stored paddy rice have been previously documented. However, the frequency and levels of mycotoxins observed in the present study varied compared to earlier findings.

For instance, the incidence and concentration of AFs and AFB2 in our study were higher than those reported by Reiter et al. [29], who found that 30% of basmati rice, whole grain rice, long grain rice, short grain rice, and puffed rice marketed in Austria were contaminated with AFs, and 1.2% with AFB2. Furthermore, Bansal et al. [30] reported the presence of AFB2 in Canadian rice with a mean concentration of 0.08 µg/kg. Similarly, lower incidences of AFG1 (1.2%) and AFG2 (2%) were reported in Ecuadorian rice [31] and AFG1 (3.33%) in Thai rice [32].

However, numerous reports have indicated that rice from Latin America, Africa, and Asia exhibits higher incidences and mean levels of AFs contamination compared to the results of this study. For example, Almeida et al. [33] reported that 45% of 166 rice samples collected from various regions in Brazil were contaminated with AFs, with an average level of 9.37 µg/kg.

Additionally, Makun et al. [34] found that 100% of stored paddy rice in Nigeria was contaminated with AFs, with concentrations ranging from 7.7 ± 1.4 to 16.5 ± 4.5 µg/kg. Similarly, polished and brown rice from Pakistan revealed contamination incidence of 41% to 56%, with mean AFs content ranging from 1.9 ± 1.20 µg/kg to 12.4 ± 0.98 µg/kg [35,36]. Furthermore, extremely high levels of AFs were reported in parboiled rice from India, with concentrations ranging from 60 to 600 µg/kg [37].

Notably, according to our previous study, AFs contamination in Mekong Delta rice was found to have a higher frequency (60%), though the average concentration was similar, ranging from 1.88 to 4.00 µg/kg, depending on the lower bound (LB) and upper bound (UB) estimates [8].

Similarly, a variety of previous studies have revealed the occurrence of AFB1 in rice across the world, including in Vietnam [38], Thailand [39,40,41], Philippines [42], South Korea [4], Japan [43,44], Pakistan [35,36], United Arab Emirates [45], India [46], and Columbia [47], with frequencies ranging from 3% to 94.8% and concentrations ranging from 0.025 to 29.82 µg/kg, consistent with the findings in our study.

In terms of FB1 contamination in rice, the incidence of this toxin was generally lower, but the mean level was higher compared to previous studies. For instance, Phan et al. [8] reported that rice from the Mekong Delta was contaminated with FBs at a frequency of 74%, with average concentrations ranging from 227 to 290 µg/kg, depending on the lower bound (LB) and upper bound (UB) estimates. Additionally, FB1 was detected in 8% to 42% of rice samples, with mean levels ranging from 0.8 ± 0.72 µg/kg to 53.2 µg/kg in Ecuador [34], Malaysia [48], Canada [30], and Pakistan [35,36].

Moreover, OTA has been frequently observed in rice from various countries worldwide, with incidences ranging from 2.9% to 100% and levels from 0.02 to 92 µg/kg in Vietnam [49], South Korea [4], white and brown rice from Pakistan [35], Brazil [28,33], Portugal [50], Morocco [51], and Côte D’ Ivore [52].

Several previous studies have indicated the presence of emerging mycotoxins in rice. For instance, ZEN was detectable in various types of Thai rice, such as rice berry, white sticky rice, GABA rice, brown rice, black sticky rice, and white rice, with incidence rates ranging from 3.33% to 46.67% [32]. BEA was observed at high levels in Thai rice [32], as well as in rice from Iran [17], Spain [51], and Morocco [53], and even in Finnish wheat [54] and Moroccan breakfast and infant cereals [55]. MON was detected in imported rice samples [56], with mean levels ranging from 8.25 to 265.3 µg/kg and incidences of 1% to 6.5%. Additionally, ENBs were found at relatively high levels in rice (30%) [53]. Notably, AFs (AFB1, AFB2, AFG1, AFG2), FB1, ZEN, OTA, and emerging mycotoxins, including BEA and ENN B, were also investigated in 125 rice bran samples from Cambodia, Laos, Myanmar, and Thailand [57].

In this study, the types, incidence, and levels of mycotoxins varied between paddy rice collected in 2018, 2019, and 2022. This variation could be attributed to differences in average temperatures and rainfall in the Mekong Delta during these years. The average temperature in 2018 and 2019 ranged from 26.6 °C to 29.9 °C, which was higher than in 2022 (26.6 °C to 28.7 °C). Additionally, rainfall in 2018 and 2019 was lower than in 2022 [24]. As a result, several emerging mycotoxins were detected in 2022, whereas AFs and OTA were predominantly observed in 2018 and 2019. Moreover, the type, concentration, and prevalence of mycotoxins differed among the six growing areas (Figure 1, Figure 2, Figure 3 and Figure 4 and Table 2), and our findings also differ from those of previous studies. This variation could be associated with various factors, including climate conditions, geographical location, agricultural practices, and the susceptibility of commodities to fungal invasion during the pre-harvest, storage, and processing measures, all of which could influence the presence and contamination levels of mycotoxins [57,58,59]. Additionally, the procedure to analyze BEA, MON, and FUS was not established for the samples collected in 2018 and 2019, whereas the detection of these mycotoxins was included in the protocol for later samples.

In this study, only ZEN was detected in rice samples collected from Can Tho, Ben Tre, and Long An. This could be attributed to the application of Trichoderma-based fertilizers by rice companies and farmers in Can Tho, which improves soil quality and inhibits mycotoxin production in the paddy [25]. In terms of rice from Ben Tre (n = 3) and Long An (n = 8), the small sample size could have contributed to the absence of other detectable mycotoxins. Therefore, future studies should consider increasing the sample size in these provinces to accurately assess mycotoxin contamination. Additionally, the milling and polishing processes might reduce mycotoxin concentrations in paddy and white rice [25].

In this study, co-occurrence of mycotoxins was observed in stored paddy rice, which is consistent with the findings of Siri-anusornsak et al. [57], who reported that rice bran samples were contaminated with four to seven mycotoxins, with incidences ranging from 2.4% to 49.6%. Co-occurrence of OTA and AFs was also observed in Brazilian rice [33]. The frequent occurrence of multiple mycotoxins in a single sample suggests complex interactions between fungal species and potentially varying environmental or storage conditions that favor the simultaneous presence of several mycotoxins. This underscores the need for comprehensive monitoring strategies that account for multiple contaminants to effectively assess and mitigate potential health risks.

## 4. Conclusions

This study indicates that mycotoxin contamination in stored paddy rice varies across different years and production areas in the Mekong Delta region, Vietnam. Some samples were found to be contaminated with levels of AFB1, OTA, and ZEN that exceeded the maximum limits set by the Vietnamese government. Additionally, several emerging mycotoxins were detected and many samples were co-contaminated from two to five mycotoxins. This underscores the need for ongoing monitoring and potentially revised storage protocols to mitigate the risk of mycotoxin contamination. As a precautionary measure, it is recommended that mycotoxin analyses are made mandatory before any production batch is distributed for sale to ensure the quality and safety of the food.

## 5. Materials and Methods

### 5.1. Study Location and Sampling

**Figure 5:**
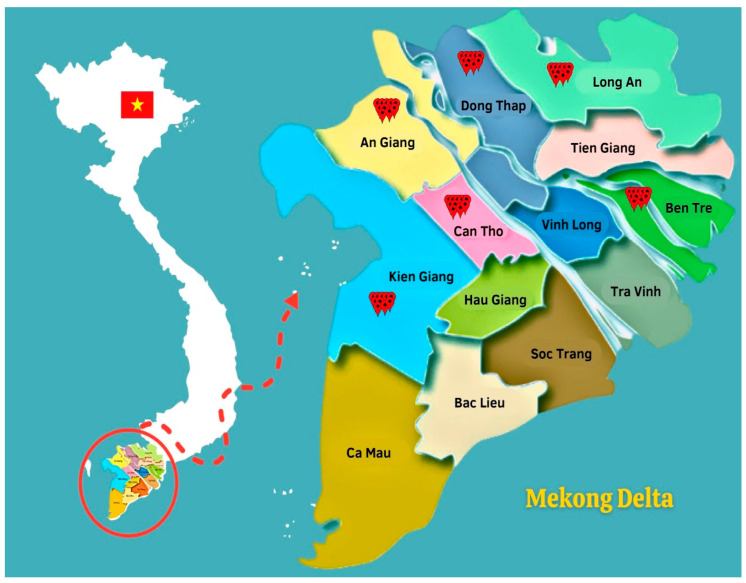
Sampling locations–Dong Thap, An Giang, Can Tho, Kien Giang, Long An, and Ben Tre provinces–in Mekong Delta, Vietnam.

A total of 96 stored paddy rice samples were collected from farmers and rice companies or cooperatives belonging to contract farming systems, characterized by a high yield production of rice and representative in six regions, namely Dong Thap, An Giang, Can Tho, Kien Giang, Long An, and Ben Tre (Figure 5) in 2018–2019 and 2022. Stored paddy samples from each farmer were collected at the warehouse and these samples were randomly taken in 10 different bags (approximate 30–50 kg/bag). Subsequently, each sample, collected in different bags, was mechanically pooled into one composite sample (2 kg) and then stored in a polyethylene zipper bag. The samples collected in 2018–2019 were transported to Ghent University, Belgium, and stored at −20 °C for mycotoxin analysis. However, paddy rice collected from 2022 was analyzed at Patent Co. Doo Misicevo, Hungary.

### 5.2. Mycotoxin Analysis by LC-MS/MS

#### 5.2.1. Extraction and Analysis of Mycotoxins in Samples Collected in 2018 and 2019

##### Mycotoxins Extraction

Each ground paddy rice sample (5 g) was spiked with de-epoxy-deoxynivalenol (DOM) at 250 µg/kg, serving as an internal standard (Biopure Romer Labs, Oostvoorne, The Netherlands). To extract the analytes, 20 mL of a solvent mixture (acetonitrile/water/acetic acid, 79/20/1, *v*/*v*/*v*) was added to the samples. This mixture was agitated for 1 h, followed by centrifugation at 4000 rpm/15 min. Afterwards, the supernatant was applied to a pre-conditioned C18 solid-phase extraction (SPE) column (Phenomenex, Utrecht, The Netherlands), and the eluate was collected in a 25 mL volumetric flask. Subsequently, 5 mL of hexane was used to re-extract the samples, yielding a defatted extract divided into two portions. The first fraction (6 mL) was filtered through a Whatman glass microfilter (VWR International, Zaventem, Belgium). The second fraction (10 mL) was combined with 20 mL of acetonitrile/acetic acid (99/1, *v*/*v*) and purified using a MultiSep^®^ 226 AflaZon column (Romer Labs, Gernsheim, Germany). After washing the column with 5 mL of acetonitrile/acetic acid (99/1, *v*/*v*), the combined eluate and 2 mL of the filtered fraction were dried under a nitrogen stream at 40 °C/1 atm. The dried residue was dissolved using 150 µL of acetonitrile/acetic acid (99/1, *v*/*v*) and centrifuged at 4000 rpm at 4 °C in 5 min. The supernatant was transferred to an Ultrafree^®^ centrifuge filter (Millipore Bedford, MA, USA) and spun at 10,000 rpm for 5 min. Finally, the filtrate was collected in a vial for LC-MS/MS analysis of mycotoxins, following the method described by Majeed et al. [36].

##### LC-MS/MS Analysis

Liquid chromatography-tandem quadrupole mass spectrometry was performed using an ultra-high performance liquid chromatography system (Waters ACQUITY, Milford, MA, USA). The system featured a symmetry guard column (3.5 µm, 10 × 2.1 mm) and a symmetry C18 analytical column (5 µm, 150 × 2.1 mm), both supplied by Waters, Zellik, Belgium. Twenty-three types of mycotoxins were analyzed, employing a Quattro Premier™ XE tandem quadrupole mass spectrometer (Waters, Milford, MA, USA). Matrix-matched calibration curves were generated using linear regression, yielding coefficients of determination (R^2^) between 0.951 and 0.999. Mycotoxin recovery rates averaged between 80% and 111%. Precision levels for intraday and interday measurements ranged from 3–14% and 6–20%, respectively. Limit of detection (LOD) values were 0.5 µg/kg for AFB1, AFB2, AFG1, and AFG2; 13 µg/kg for FB1; 11 µg/kg for AME; and 0.75 µg/kg for OTA. Corresponding limits of quantification (LOQ) were 1.5 µg/kg for each AF, 26 µg/kg for FB1, 21 µg/kg for AME, and 2.5 µg/kg for OTA [36,60].

##### Method Validation

The validation parameters were determined based on the relative peak area, using deepoxy-deoxynivalenol (DOM) as the internal standard for NIV, DON, Fusarenon-X (FUS-X), Neosolaniol (NEO), 3-acetyl-deoxynivalenol (3-ADON), 15-acetyl-deoxynivalenol (15-ADON), HT2, T2, and DAS, while Zearalanone (ZAN) was used as the internal standard for other mycotoxins. The parameters assessed included linearity, limit of detection (LOD), limit of quantification (LOQ) (Table A1), apparent recovery (%), intraday and interday repeatability, and expanded measurement uncertainty (Table A2). No interfering peaks were observed at the retention times of the targeted mycotoxins, ensuring specificity. Specificity was further verified by analyzing 20 blank rice samples using LC–MS/MS, with spiked calibration levels for each mycotoxin evaluated in triplicate over four validation days (Table A2). For contamination levels outside the validated range, alternative calibration curves were generated. The method-matched calibration curves, fitted by linear regression, demonstrated a coefficient of determination (R^2^) ranging from 0.991 to 0.999. Mean recoveries for all mycotoxins ranged from 80 to 111% (Table A2), with relative standard deviations for intraday (RSDr) and interday (RSDR) repeatability ranging from 3 to 14% and 6 to 20%, respectively. LOD values ranged from 0.5 to 53 µg/kg, while LOQs were 1.5 to 105 µg/kg. Expanded measurement uncertainty was calculated, ranging from 2.01 to 34.4%. The LOD and LOQ calculations were based on method-matched calibration curves [36]. Additionally, ion ratios for qualifier and quantifier ions met EU criteria, confirming the positive identification of compounds. Overall, the validation results for this multi-mycotoxin method complied with the European Commission Performance Criteria [61].

#### 5.2.2. Extraction and Analysis of Mycotoxins in Samples Collected in 2022

##### Mycotoxins Extraction

Each ground paddy rice sample (5.0 ± 0.1 g) was put into a 50 mL conical tube. Subsequently, 20 mL of extraction solvent (acetonitrile/water/formic acid, 80/19/1, *v/v/v*) was added to the samples, and the mixture was shaken on an orbital shaker at 250 rpm for 90 min at room temperature. After shaking, the mixture was centrifuged at 4200 rpm for 5 min, and then 450 μL of the supernatant was transferred to a glass vial. Then, 750 μL of ultrapure water (18.2 MΩ/cm) was added to the supernatant, and the mixture was vortexed thoroughly. In the next step, the mixture (1200 µL) was filtered using a membrane syringe filter (Nylon Syringe Filter, pore size 0.22 µm) into a 2 mL glass vial. Finally, the filtrate was analyzed for mycotoxins using LC-MS/MS.

Regarding the spiking procedure, 5 g of homogenized and finely ground sample was placed into a 50 mL conical tube, and 200 µL of a multi-mycotoxin spiking solution was added to the sample. The extraction steps were carried out in the same manner as described above for the sample extraction

##### LC-MS/MS Analysis

Liquid chromatography-tandem quadrupole mass spectrometry (LC-MS/MS) was conducted using an analytical column (Waters CORTECS^®^ UPLC C_18_, 2.1 × 100 mm, 1.6 µm) paired with a guard column (Waters CORTECS^®^ UPLC C_18_ VanGuard™, 2.1 × 5 mm, 1.6 µm). The system was equipped with an Agilent Technologies 1290 series II auto liquid sampler with a thermostat and a column oven. The solvent delivery system was operated using the Agilent Technologies 1290 series II Flex pump. Mass spectrometric detection was performed using an Agilent Technologies 6460 c MS/MS with a Jet Stream electrospray ion source.

The injection volume was set to 5 µL, and the column temperature was maintained at 50 °C. The MS/MS parameters were as follows: gas temperature of 200 °C, gas flow of 8 L/min, nebulizer pressure of 40 psi, sheath gas temperature of 350 °C, sheath gas flow of 11 L/min, capillary voltage of 3500 V, and nozzle voltage of 500 V.

The average recoveries for all the mycotoxins ranged from 80% to 109%. The limits of detection (LOD) for the mycotoxins were as follows: 1 µg/kg for ENB; 3 µg/kg for FUS and MON; 5 µg/kg for ZEN; 25 µg/kg for FB1. The LC-MS/MS analysis was conducted with the gradient settings outlined in Table A3.

## Figures and Tables

**Table 1:** The prevalence of co-occurrence of mycotoxins in stored paddy rice collected in 2022 (n = 50).

Co-Occurence	The Combination of Mycotoxins	N° of Sample (%)
2 mycotoxins	Fusaric acid + Beauvericin	1 (2)
	Zearalenone + Fusaric acid	1 (2)
3 mycotoxins	Zearalenone + Fusaric acid + Beauvericin	1 (2)
	Moniliformin + Fusaric acid + Beauvericin	2 (4)
4 mycotoxins	Zearalenone + Fusaric acid + Beauvericin + Enniatin B	1 (2)
	Zearalenone + Moniliformin + Fusaric acid + Beauvericin	3 (6)
5 mycotoxins	Zearalenone + Fumoninin B1 + Moniliformin + Fusaric acid + Beauvericin	1 (2)

**Table 2:** The mycotoxin concentration (µg/kg) in stored paddy rice collected in Mekong Delta, Vietnam in three years (2018, 2019 and 2022).

Mycotoxin	Dong Thap (n = 25)		Kien Giang (n = 10)		An Giang (n = 24)		Long An (n = 8)		Ben Tre (n = 3)		Can Tho (n = 26)	
	Mean ± SD	Range	Mean ± SD	Range	Mean ± SD	Range	Mean ± SD	Range	Mean ± SD	Range	Mean ± SD	Range
Zearalenone	82.5 ± 97.2	31.1–255	67.5	67.5	27.8 ± 14.4	9.8–45.8	67.5 ± 56.5	25.1–166	22.8 ± 3.7	20.1–25.4	35.3 ± 29.7	14.1–85.1
Fumonisin B1	689	689	33.5 ± 20.0	20.3–72	-	-	-	-	-	-	-	-
Fusaric acid	66.3 ± 87.8	4.2–128.3	19.9 ± 15.2	5–48.9	-	-	-	-	-	-	-	-
Moniliformin	9.1	9.1	8.2 ± 3.3	4.9–13.4	-	-	-	-	-	-	-	-
Beauvericin	2.8 ± 2.3	1.2–5.4	7.0 ± 4.0	1.4–1.3	-	-	-		-		-	-
Enniatin B	-	-	1.6	1.6	-	-	-	-	-	-	-	-
Aflatoxin B1	-	-	-	-	12.1 ± 3.5	10–16.1	-	-	-	-	-	-
Aflatoxin B2	2.2 ± 0.1	2.1–2.2	-	-	3.3 ± 1.5	2.2–4.3	-	-	-	-	-	-
Aflatoxin G1	-	-	-	-	3.1 ± 0.1	3.0–3.3	-	-	-	-	-	-
Aflatoxin G2	-	-	-	-	6.5 ± 1.4	5.5–7.4	-		-		-	-
AME	17.8	17.8	-	-	-	-	-	-	-	-	-	-
Ochratoxin A	-	-	-	-	38.2	38.2	-	-	-	-	-	-

(-): no detection; AME: Alternariol Monomethyl Ether; n: number of sample.

## Data Availability

The original contributions presented in this study are included in the article. Further inquiries can be directed to the corresponding author.

## Appendix A

**Table A1:** Limits of detection (LOD) and limits of quantification (LOQ), linearity range, and coefficient of determination (R^2^) of all the measured mycotoxins in rice [36].

Mycotoxin	LOD (µg/kg)	LOQ (µg/kg)	Range (µg/kg) R^2^	
Zeralenone (ZEN)	7	13	37.5–150	0.998
Enniatin B (ENNB)	50	100	100–400	0.992
Roquefortine-C (ROQ-C)	4	8	10–40	0.993
Sterigmatocystine (STERIG)	9	18	25–100	0.995
Alterbariol methylether (AME)	11	21	100–400	0.991
Fumonisin B2 (FB2)	17	35	100–400	0.993
Ochratoxin A (OTA)	0.75	2.5	2.5–20	0.994
Fumonisin B3 (FB3)	53	105	125–500	0.995
T-2 toxin (T2)	22	44	50–200	0.993
Fumonisin B1 (FB1)	13	26	100–400	0.994
HT-2 toxin (HT2)	13	26	50–200	0.996
Alternariol (AOH)	22	44	50–200	0.997
Diacetoxyscirpenol (DAS)	3	6	10–40	0.996
Aflatoxin B_1_ (AFB_1_)	0.5	1.5	2.5–20	0.994
Aflatoxin B_2_ (AFB_2_)	0.5	1.5	2.5–20	0.995
Aflatoxin G_1_ (AFG_1_)	0.5	1.5	2.5–20	0.996
Aflatoxin G_2_ (AFG_2_)	0.5	1.5	2.5–20	0.994
15-Acetyl-deoxynivalenol (15-ADON)	9	19	25–100	0.995
3-Acetyl-deoxynivalenol (3-ADON)	16	33	50–200	0.998
Fusarenon-X (FX)	9	18	100–400	0.993
Neosolaniol (NEO)	12	23	50–200	0.996
Deoxynivalenol (DON)	19	37	100–400	0.992
Nivalenol (NIV)	8	16	50–200	0.999

**Table A2:** Intraday repeatability (RSDr), interday repeatability (RSDR), apparent recovery (%), and expanded measurement uncertainty (U) of the individual mycotoxins for the rice matrix analyzed at spiked concentration levels, in triplicate on four different days [36].

Mycotoxin	Spike Conc.	RSDr	RSDR	Apparent	Expanded Measurement
	(µg/kg)	(%)	(%)	Recovery (%)	Uncertainty U (%) *
ZEN	37.5	8	14	100	6
	56.3	4	16	99	3
	75	6	12	100	5
	100	5	9	100	3
	150	4	7	100	2
ENNB	100	14	20	80	17
	150	12	17	86	17
	200	9	17	111	13
	300	12	14	96	24
	400	10	12	99	22
ROQ-C	10	9	16	111	34
	15	8	14	99	19
	20	5	14	93	14
	30	3	12	98	3
	40	3	8	102	4
STERIG	25	13	19	110	16
	37.5	11	17	97	12
	50	8	14	94	8
	75	5	15	99	5
	100	4	12	101	3
AME	100	11	12	102	9
	150	9	12	99	4
	200	6	11	99	3
	300	7	10	99	3
	400	5	9	100	3
FB2	100	9	16	98	5
	150	7	13	100	3
	200	6	11	100	4
	300	6	9	99	2
	400	5	8	100	2
OTA	2.5	14	19	101	14
	5	11	17	99	5
	10	9	13	100	11
	15	8	14	99	4
	20	5	11	100	2
FB3	125	7	16	99	3
	187.5	8	14	99	3
	250	6	11	100	4
	375.5	5	8	99	3
	500	4	7	99	3
T2	50	13	18	106	30
	75	10	15	96	17
	100	11	14	99	9
	150	11	14	99	7
	200	9	13	100	6
FB_1_	100	9	18	99	3
	150	8	16	100	2
	200	5	12	100	3
	300	4	9	99	3
	400	4	8	100	2
HT2	50	14	20	105	26
	75	13	19	98	19
	100	9	17	97	7
	150	6	13	100	3
	200	3	12	100	4
AOH	50	9	19	101	18
	75	9	17	99	17
	100	7	15	98	16
	150	6	11	101	14
	200	3	8	99	9
DAS	10	7	16	98	21
	15	5	15	102	17
	20	6	11	99	11
	30	6	9	99	8
	40	4	7	100	4
ABF_1_	2.5	8	15	95	16
	5	7	13	100	16
	7.5	7	11	96	11
	10	5	9	100	9
	15	4	7	102	7
	20	4	7	98	4
ABF_2_	2.5	7	17	96	14
	5	7	15	100	12
	7.5	5	13	97	11
	10	5	12	101	5
	15	4	9	100	3
	20	3	7	99	4
AFG_1_	2.5	7	18	98	13
	5	6	18	101	12
	7.5	5	15	99	11
	10	4	12	101	7
	15	3	9	98	7
	20	3	6	100	5
AFG_2_	2.5	8	16	97	14
	5	7	15	101	13
	7.5	6	13	99	11
	10	5	13	100	7
	15	5	8	99	5
	20	4	7	100	4
15-ADON	25	13	15	86	7
	37.5	11	11	84	6
	50	8	11	84	5
	75	6	9	85	3
	100	4	9	85	3
3-ADON	50	12	20	102	9
	75	11	16	99	77
	100	9	16	98	6
	150	6	14	100	3
	200	5	11	100	2
FX	100	13	19	100	8
	150	11	17	100	5
	200	9	17	99	4
	300	7	13	99	3
	400	5	12	100	3
NEO	50	9	17	100	9
	75	7	17	99	7
	100	6	15	100	6
	150	4	11	99	4
	200	5	9	100	3
DON	100	7	15	102	8
	150	8	13	99	6
	200	6	10	99	5
	300	5	9	100	4
	400	3	6	100	3
NIV	50	7	16	99	5
	75	6	14	99	4
	100	7	13	100	3
	150	5	11	99	3
	200	4	8	100	3

* With a maximum error limit of 20% and a β-tolerance interval of 0.85; ZEN: Zearalenone; ENNB: Enniatin B; ROQ-C: Roquefortine-C; STERIG: Sterigmatocystine; AME: Alternariol Methylether; FB1, FB2, and FB3: Fumonisin B1, B2, and B3; OTA: Ochratoxin A; T2: T-2 toxin; HT2: HT-2 toxin; AOH: Alternariol; DAS: diacetoxyscirpenol; AFB1, B2, G1, and G2: Aflatoxin B1, B2, G1 and G2: 15-ADON: 15-acetyl-deoxynivalenol; 3-ADON: 3-acetyl-deoxynivalenol; F-X: Fusarenon-X; NEO: Neosolaniol; DON: Deoxynivalenol; NIV: Nivalenol.

**Table A3:** Gradient setting for LC-MS/MS condition for analysis of mycotoxins in paddy collected in 2022.

Time (min)	Mobile Phase				Flow (mL/min)
	A (%)	B (%)	C (%)	D (%)	
0	0	0	97	3	0.3
3.0	0	0	97	3	0.3
3.1	82	18	0	0	0.3
4.0	80	20	0	0	0.3
9.0	80	20	0	0	0.3
15.0	20	80	0	0	0.3
17.0	20	80	0	0	0.3
18.0	0	100	0	0	0.3
21.0	0	100	0	0	0.3
22.0	0	0	97	3	0.3

A: 0.1 mL formic acid (LC-MS grade), 0.32 g ammonium formate (LC-MS grade) and 1000 mL H_2_O; B: 0.1 mL formic acid (LC-MS grade), 0.32 g ammonium formate (LC-MS grade); 750 mL MeOH (LC-MS grade) and 250 mL acetonitrile (LC-MS grade); C: 0.5% acetic acid (ACS) in ultrapure water (18.2 MΩcm^−1^). D: acetonitrile (LC-MS grade).
